# Integrating Interactive Web-Based Technology to Assess Adherence and Clinical Outcomes in Pediatric Sickle Cell Disease

**DOI:** 10.1155/2012/492428

**Published:** 2012-06-04

**Authors:** Lori E. Crosby, Ilana Barach, Meghan E. McGrady, Karen A. Kalinyak, Adryan R. Eastin, Monica J. Mitchell

**Affiliations:** ^1^College of Medicine, University of Cincinnati, Cincinnati, OH 45221, USA; ^2^Behavioral Medicine and Clinical Psychology, Cincinnati Children's Hospital Medical Center, Cincinnati, OH 45229, USA; ^3^Department of Psychology, University of Cincinnati, Cincinnati, OH 45221, USA

## Abstract

Research indicates that the quality of the adherence assessment is one of the best predictors for improving clinical outcomes. Newer technologies represent an opportunity for developing high quality standardized assessments to assess clinical outcomes such as patient experience of care but have not been tested systematically in pediatric sickle cell disease (SCD). The goal of the current study was to pilot an interactive web-based tool, the Take-Charge Program, to assess adherence to clinic visits and hydroxyurea (HU), barriers to adherence, solutions to overcome these barriers, and clinical outcomes in 43 patients with SCD age 6–21 years. Results indicate that the web-based tool was successfully integrated into the clinical setting while maintaining high patient satisfaction (>90%). The tool provided data consistent with the medical record, staff report, and/or clinical lab data. Participants reported that forgetting and transportation were major barriers for adherence to both clinic attendance and HU. A greater number of self-reported barriers (*P* < .01) and older age (*P* < .05) were associated with poorer clinic attendance and HU adherence. In summary, the tool represents an innovative approach to integrate newer technology to assess adherence and clinical outcomes for pediatric patients with SCD.

## 1. Introduction

Sickle cell disease (SCD) is a genetic red blood cell disorder characterized by the sickling of red blood cells resulting in pain episodes, organ damage, risk for infections, and decreased life expectancy [1]. Care guidelines for SCD recommend that patients attend routine clinic appointments one to two times per year and more frequently if there are complications or if clinical monitoring is needed to assess tolerance to medications and other treatments [1, 2]. Preventative care such as flu shots, immunizations, and monitoring labs is also essential to effectively manage sickle cell disease [3]. Hydroxyurea (HU), which is used to increase fetal hemoglobin (Hb F), has been shown to decrease morbidity and mortality in patients with SCD [4]. Studies have indicated that daily oral HU use is associated with reduced pain crises, hospitalizations, acute chest syndrome, and transfusions and improved growth and health-related quality of life [5–9]. Thus, the consequences of nonadherence to clinic attendance and HU treatments result in increased morbidity [10], healthcare costs [11], and decreased quality of life [12].

There is limited research available examining treatment nonadherence in pediatric SCD. A meta-analytic review found a nonadherence rate for clinic appointments in pediatric populations of approximately 40% [13]. Similarly, nonadherence rates for clinic appointments in SCD range between 36%–44% [14, 15]. On the surface, it would seem that patients who attend their appointments would demonstrate higher levels of adherence to their treatment regimen; however, the relationship between clinic attendance and treatment adherence appears to be complex. As an example, Finney et al. [16] found that the 48% of patients who kept their follow-up appointment had been nonadherent to their prescribed regimen. Thus, it is important to assess barriers to the treatment regimen even with patients who attend appointments regularly.

Research on medication adherence in SCD suggests higher rates for acute medications than daily medications. Dampier et al. [17] found that 85% of adolescents with SCD took analgesic medications on days when they experienced sickle-cell related pain. In contrast, studies found that 38%–60% of pediatric and adult patients were adherent to their prescribed days of home chelation therapy or deferoxamine usage [18, 19] and rates ranged from 12%–67% for young children with SCD taking prophylactic penicillin [20–22]. Studies of HU adherence in SCD have typically utilized small sample sizes or single measures of adherence (e.g., pill counts) [23]. Overall, these studies indicate variable rates of adherence and lower rates for long-term trials [23, 24]. For example, Zimmerman and colleagues discontinued HU in 12% of their participants due to nonadherence [24]. Data from our own clinic indicate that 30% of patients who were prescribed HU were discontinued due to nonadherence. Poor adherence with HU may have unintended consequences as the medication can be discontinued on the assumption that the patient is a nonresponder, while other patients may have their dose increased to a level that is toxic when they actually begin taking it. Standardized and multidimensional approaches to measuring adherence are needed to ensure that patients receive optimal benefits from the medication while also minimizing risks to patients.

Overall, research indicates low and variable adherence rates to different components of the SCD treatment regimen. Studies identifying barriers to adherence have indicated a clear link between adherence and poor clinical outcomes in pediatric populations [25]. In pediatric SCD samples, studies have identified the following as key barriers: competing activities, health status, patient-provider relationships, adverse clinical experiences, and forgetting [26]. Other studies have identified sociocultural barriers to adherence including developmental factors, transportation, and health literacy [27–29]. Multicomponent, behavioral, and educational interventions to promote adherence have been found to be well-established pediatric treatments, [30, 31]; however, a recent study indicates that the quality of the adherence assessment is one of the best predictors for improving clinical outcomes [25]. Newer technologies (e.g., computer-based, text messaging) represent an opportunity for developing high quality, standardized and cost-effective assessments of treatment adherence and clinical outcomes such as patient experience of care (e.g., communication with and responsiveness of staff, quality of information received, wait time, satisfaction, etc.), disease-specific outcomes (e.g., labs) and health-related quality of life [32]. These types of approaches have been used effectively in chronic illness populations including adults with hypertension, diabetes [33, 34], and pediatric asthma [35].

The current study represents a first step in integrating interactive web-based technologies in SCD clinical care. The aims of the current study were to pilot an interactive family-based web-based tool, the Take-Charge Program, to assess adherence and clinical outcomes including: (1) patient HU adherence, barriers to HU adherence, and potential solutions to improve HU adherence; (2) patient clinic attendance adherence, barriers to clinic attendance adherence, and potential solutions to improve clinic attendance; and (3) clinical outcomes (patient experience of care, sickle-related outcomes hemoglobin level, ANC, MCV, and percent fetal hemoglobin level for HU patients).

## 2. Participants and Methods

Data presented in this paper are from the baseline assessment of a larger longitudinal study being conducted at a tertiary urban pediatric medical center in the Midwest. Eligible participants were patients of a comprehensive SCD clinic, age 6 to 21 years (and their caregivers), and prescribed hydroxyurea (HU) therapy or referred by clinic staff for attendance problems. Patients who had significant health complications (e.g., acute illness, recent stroke) that would interfere with the completion of the study or significant cognitive or developmental disabilities were excluded due to the demand on participants to understand questions in the assessment. Of the 182 patients in the clinic, 98 were eligible based on the above criteria. To date, 47 patients have been enrolled in the study with 4 being withdrawn because they no longer met criteria; thus, data will be presented on the 43 participants in the sample. Potential participants were identified by the clinical staff and the research team confirmed eligibility criteria. All participants were approached during a scheduled clinic visit. After obtaining consent, data collection proceeded at that visit.

### 2.1. Measures

Patients and caregivers completed the following measures.

#### 2.1.1. Background Information Form

This form summarized personal/family demographic information, including participant school/vocational history, parent education, family income, family transitions, and life events. In addition, self-report of pain frequency and intensity, and hospital and emergency room visits over the past year was collected for comparison with data collected from the medical record review.

#### 2.1.2. Barriers to Care Questionnaire [36]

This validated and reliable 40-item questionnaire measures parents' report of encounters or situations that may interfere with access to care, use of care, the patient-physician experience, or adherence with medical instructions. Barriers are conceptualized as multidimensional and include pragmatics (logistics, cost), expectations about care, health knowledge and beliefs, marginalization and health care navigation skills.

#### 2.1.3. The Take Charge Program (Web-Based Tool)

A voice-automated interactive web-based assessment tool was developed based on questions from the Disease Management Interview [37] and consultation from clinic staff. Caregiver and child dyads complete the tool which takes approximately 15–20 minutes. The measure included questions and prompts that enabled patients and caregivers to identify barriers to adherence to clinic attendance and HU from a standard list [26, 37] and at least one strategy for improving adherence. The voice-automation increased the validity as literacy was not required to complete the measure. The initial development of the Take-Charge Program has been described elsewhere [38]. During the second phase of development, patients and caregivers matched barriers and potential solutions for HU. Cognitive interviewing was used to ensure that questions were being understood as intended [36, 38]. The web-based tool assessed clinic attendance, hydroxyurea adherence, and patient experience of care. The Clinic Attendance Module assessed self-reported adherence to clinic appointments on a 10-point Likert-type scale with 10 representing perfect adherence. The hydroxyurea module assessed self-reported HU adherence on a 10-point Likert-type scale with 10 representing perfect adherence. Both modules also asked participants to select applicable barriers and a potential solution to address these barriers. The Patient Experience of Care Module, an adapted version of Krahn et al.'s [39] questionnaire using a 4-point Likert scale and 2 open-ended questions assessed (1) wait time in clinic, (2) understanding of treatment recommendations by healthcare team, (3) time spent with healthcare team, and (4) helpfulness of web-based tool. Once participants identified a potential solution to try, clinic staff or a member of the research team utilized a standardized problem-solving intervention adapted from Behavioral Family Systems Therapy [40] to help participants develop a specific plan to implement the solution.

#### 2.1.4. Medical Record Review

Electronic medical records were reviewed to confirm participant's type of SCD and collect the following data: hemoglobin level, ANC, MCV, percent fetal hemoglobin, clinic attendance, ER visits, and hospitalizations. Information on the participant's prescribed treatment plan was also collected and verified with clinic staff.

### 2.2. Data Analysis

 Data collected from the web-based tool and electronic medical record (EMR) were integrated into a single database. Descriptive statistics and frequencies were utilized to summarize demographics, health characteristics, health care utilization, self-reported adherence, barriers, potential solutions, and patient experience of care. Pearson product-moment correlation coefficients were computed to conduct exploratory analyses to assess the relationship between adherence to clinic visits (e.g., self-reported barriers on the Take-Charge Program, number of barriers), adherence to HU (e.g., self-reported barriers on the Take-Charge Program, number of barriers), demographics, patient experience of care and sickle cell related outcomes. All analyses were conducted in IBM SPSS Statistics 19 (SPSS: An IBM Company).

## 3. Results

### 3.1. Participants

Study participants included 43 youth with SCD (*M* = 12.81 + 3.98 years; 39.5% Male; 79.0% HbSS; 9.3% HbSC; 4.7% H*β* ^+^Thal; 7.0% other) and their primary caregivers (83.7% mothers; 7.0% fathers; 9.3% other). Additional demographic characteristics are reported in Table 1. This sample is representative of the total SCD clinic population, with the exception of hemoglobin type (fewer participants with HbSC) but is consistent with the fact that the majority of participants in the study were on HU therapy. With respect to clinical characteristics, 62.2% of the sample reported having six or less pain days in the past 12 months, and 61.5% reported missing 6 days of school for pain in the past 12 months. Emergency room (ER) visit and hospitalization data indicated that most participants had three or fewer ER visits (95.1%) or hospitalizations (90.5%) in the past 12 months (see Table 2).

### 3.2. Clinic Attendance

According to the data, approximately half of participants (47.5%) indicate that they “always come” when describing their clinic attendance over the previous 12 months. This self-reported adherence was similar to data obtained from the EMR which indicated that 55% of patients never missed an appointment during this same period. Although about half of patients missed at least one appointment, a higher percent (75%) understood that their SCD providers recommended clinic appointments at least twice a year, and participants attended 84.5% of all scheduled appointments. Top-rated barriers on the Barriers to Care Questionnaire (BCQ) were related to pragmatics (i.e., logistics, cost) (*M* = 76.1; SD = 15.7) and health-care navigation skills (*M* = 79.4, SD = 22.4). When asked specifically about barriers to clinic attendance on the Take-Charge Program, participants reported the following barriers: transportation difficulties (22.5%), inability to take off from work/school (17.5%), forgetting (10%), waiting too long (7.5%), competing activities (e.g., sports; 5%), feeling tired (5%); dislike of treatments (2.5%), feeling it is unnecessary (2.5%), and other (10%). Other barriers included getting appointment dates and times confused, not having transportation vouchers/setup, and not having appointments available at a time that works with the family's schedule. For potential solutions, 47.1% chose an individualized solution; 29.4% reported that they would try scheduling their appointment at a better/different time; and 5.9% reported that they would try setting an alarm (e.g., phone).

Participants were asked to rate their adherence on a scale of 0 to 10 with 10 being no problems with adherence. The mean rating for the sample was 8.9/10 (*N* = 40; SD : 2.1). They were also asked to rate how many visits they miss per year on average. The mean for missed visits was 1.0 (*N* = 40; SD = 1.4). Exploratory analyses showed that a greater percentage of no shows over the 12 month period prior to study enrollment was positively related to (1) more self-reported barriers to clinic attendance on the Take Charge Program *r* = .550, *n* = 40, *P* < .001; (2) the number of visits required *r* = .455, *n* = 39, *P* < .01; (3) age *r* = .317, *n* = 41, *P* < .05; and (4) marginally related to satisfaction with clinic visits *r* = .323, *n* = 40, *P* < .052.

### 3.3. Hydroxyurea Adherence

 While less than a third of participants (26.7% saying “yes”) reported that they “always took their HU,” overall, participants rated themselves as an 8.8 on a 10-point scale for “how often do they take their medicines?” which converts to 88%. When asked about missing doses, participants reported that they missed an average of 1.3 doses of HU per week. Participants reported the following barriers to HU on the Take-Charge Program: forgetting (56.7%), not having the medication with me (26.7%), the medications running out (23.3%), yucky taste or smell (12.5%), upset stomach (12.5%), and being not sure why I take it (3.1%). Other barriers reported included not wanting to stop what the patient is doing to take medication and taking too many medications. For potential solutions, 20% chose to try putting the medication next to something they do every day (e.g., toothbrush, breakfast table); 15% chose to use an alarm clock or cell phone alarm; 15% chose to use a pill box; and 15% chose to use a calendar. Additional solutions selected were reminder calls, and coordinating better with child/caregiver. Also, clinic staff rated that approximately 40% of participants were adherent to medications based on clinical data but staff felt that the clinical data of the other 60% indicated nonadherence or that further monitoring was needed. Number of missed doses of HU during the previous two weeks was related to age, with older age being related to greater nonadherence *r* = .372, *n* = 31, *P* = .036 and greater number of barriers reported on the Take-Charge Program *r* = .421, *n* = 31, *P* = .023.

### 3.4. Clinic Integration

Nearly all participants and their parents (41/43) completed the web-based assessment tool while waiting to see the care team for their appointment. The tool collected accurate and complete data with minimal missing data (3 participants due to technical errors). The majority of participants (64.9%) rated the web-based tool as very helpful and another twenty-five percent (24.3%) rated it as a little helpful.

### 3.5. Clinical Outcomes

**Patient Experience of Care**

The majority of patients (82%) reported a reasonable wait time (43% not at all; 29% short time) and only 18% reported that their wait time for the visit was too long. With respect to the visit itself, approximately 79.5% of participants reported that they were satisfied with the amount of time the medical team spent with them during their visit. In addition, the majority of participants (82.1%) reported that what the medical team shared with them was very helpful. Specifically, participants reported that the medical information was helpful (16%), and they found discussions about the treatment plan (e.g., discussion around medicine dosage and test results; about steps she needs to take to stay healthy) very beneficial (12%). Some participants (38%) also reported that other things made the visit positive (e.g., toys, movies).

**Sickle Cell-Related Lab Values**

Lab values from the date of enrollment into the study (or within 30 days of enrollment) were obtained from an electronic portal that pulls data from the electronic medical record system. The mean hemoglobin level for participants was 9.7 (*N* = 39; SD = 1.28). For those participants on HU therapy, the mean percent fetal hemoglobin was 23.1 (*N* = 26; SD = 15.6), the MCV was 98.0 (*N* = 28; SD = 13.9), and the ANC was 4.2 (*N* = 27; SD = 2.44). There was not a significant relationship between lab values and patient satisfaction with the amount of time spent with the medical team or the helpfulness of the medical information shared during the visit.

## 4. Discussion

 This study highlights the potential to efficiently integrate interactive web-based technology in a clinic-based setting to assess treatment adherence, patient experience of care, and disease-specific outcomes in pediatric SCD. This study is significant as it piloted an innovative and high quality assessment process for capturing adherence data, including the barriers to adherence and potential solutions for addressing these barriers. The data from the tool showed a number of interesting trends. First, the tool proved to be a useful means for collecting data to understand adherence to clinic visits. Self-reported adherence to clinic visits was consistent with data from patients' EMR as both sources revealed a 12-month clinic attendance adherence rate (“always coming” and “attending all visits”) of approximately 50%. It should be noted that the number of missed visits and adherence may be relative given that patients had 2 to 17 visits scheduled over the course of the year based on the complexity of treatments and disease-related complications (e.g., hospital discharge followup). Taken together, these findings suggest that nearly 85% of all scheduled clinic visits were attended by participants in this study. Understanding barriers to nonadherence was also important, especially given that adherence to clinic visits does not take into account cancellations, same day cancellations or rescheduled visits as a nonattended visit. Finally, data from the Take-Charge Program was integrated with patients' EMR data providing a wealth of data to inform clinical practice in “real time.”

The barriers to clinic attendance endorsed by participants were multifaceted and included logistical (transportation, getting off work), health care navigation skills (using calendars to manage multiple appointments and medications), socioeconomic (lack of insurance), and disease-related barriers (did not feel well). BCQ mean scores for this sample were consistent with mean scores for other pediatric populations with similar challenges (e.g., asthma, children with special health care needs) [36, 41]. Participants also identified potential solutions for improving adherence and attendance (which is the basis for a larger longitudinal intervention study). Additional potential risk factors for no-shows emerged from exploratory analysis of the data which found that nonadherence to clinic visits increased with age, more required visits, and self-reported barriers to attendance. These findings, though preliminary, further highlight the richness of the data and provide meaningful trends and a basis for prioritizing patients who may be in need of additional clinical supports to ensure patient engagement and attendance.

The Take-Charge tool was also piloted to better understand the subset of patients on HU (74% of the sample). Anecdotally, nonadherence to HU is cited as a problem for many children and adolescents with SCD although developmentally-appropriate approaches for children and adolescents are very limited. The findings support that the Take-Charge tool was useful for assessing relevant information from participants on HU related to their perceptions of how they are to be taking their medications, concerns about side-effects and other barriers. Data from the tool highlighted that approaches to working with patients around medication management will need to address organizational issues [18, 19] such as helping patients and parents use calendars, phone alarms, and emerging innovative technologies (e.g., pill cases that glow in the dark and beep) to reduce the potential for “forgetting” to take medications, getting them refilled, and packing them when away from home.

Qualitative data from participants further highlight the importance of implementing family-based strategies and the need to tailor them appropriately to individual needs. Medication side-effects (e.g., taste, smell, upset stomach) and lack of awareness (i.e., to address patients who are not sure why they are taking medication) are other important barriers to address. Interestingly, some of the reasons that are commonly considered for nonadherence (e.g., the probability for loss of hair, stigma, fear of blood draws, and that the medicine would not make a difference) were not endorsed by these participants but still may be important for patients who do not agree to try the medication or who show early signs of nonadherence. Several participants noted that transportation and lack of insurance were barriers not only to clinic attendance, but also to HU adherence, highlighting the pervasiveness of income and access to health care on adherence. The barriers that are assessed in the tool appear to have some clinical utility as more barriers were related to a higher number of missed doses of medicine during the previous two weeks prior to the study. This study also supports previous research which suggests that age should also be considered as a target in the clinical evaluation, given the potential for nonadherence to medication increased with age [10, 34]. Data support the potential for the tool to reliably assess adherence and other health utilization outcomes while fostering individualized and family-based solutions for addressing barriers to adherence to clinic visits and medication. Patient experience of care data on the clinical integration of the tool showed positive trends with at least 90% of participants endorsing satisfaction/helpfulness of the tool. It was positive that satisfaction with the clinic visit was also not compromised with >90% also endorsing satisfaction with the clinic visit.

The limitations of the study should be noted. First, given the pilot/feasibility nature of the study, only patients who were engaged in our clinic were included in the study. It will be important in future clinical research to understand and address the barriers of patients (perhaps via the web or other engaging methods) who have lost contact or who are unable to attend clinic because of barriers and risks to determine if more significant intervention is needed. Second, the study included a range of participants across a broad developmental level. In addition, since this was a family-based assessment and intervention study, individual reports of barriers for patients and caregivers were not collected. This limitation should be overcome in future studies by insuring that older patients identify individual barriers to treatment adherence. Third, patients were diverse in income, disease severity, and other factors. Replicating this study with a larger sample to better understand adherence within developmental and disease-related subgroups will be important. Fourth, data collection from the EMR was a challenge in this study as corresponding lab values were not available for all study visits. It will be essential to better coordinate lab draws and study visits and to ensure study funds to pay for corresponding labs so that data can be tracked over time. Finally, the data reported here are cross-sectional and some are self-reported in nature. Future research is needed to monitor clinical outcome data longitudinally and to assess the relationship between adherence and clinical outcomes over time.

A next step in advancing our research is to refine the tool. There were a number of “other” responses that received a high endorsement which justify some additional revision of existing screens to include additional response options. While this program has the potential to be used as a tool to help improve adherence and clinical outcomes during follow-up visits, a goal and a challenge will be to maintain high patient satisfaction with the program. As the program becomes further standardized, another goal will be to streamline the clinic integration process and to pilot it in other SCD clinic settings. In spite of these limitations the study's overall goal was met which was to integrate the web-based tool within a clinic-based setting (rather than in a nonclinical research setting) and to assess adherence and clinical outcomes for pediatric patients with SCD.

## Figures and Tables

**Table 1:** Patient demographics.

	*N* (%)	Mean (SD)
Hemoglobin type		
HbSS	34 (79.0)	
HbSC	4 (9.3)	
Hb^+^Thal	2 (4.7)	
Other	3 (7.0)	
Gender		
Male	17 (39.5)	
Female	26 (60.5)	
Age		12.81 (3.98)
Race		
African-American	43 (100)	
Grade in school, median		6th
Primary caregiver		
Mother	36 (83.7)	
Father	3 (7.0)	
Other relative	4 (9.3)	
Highest grade completed by caregiver		
High school	15 (34.9)	
Some college	14 (32.6)	
College degree	13 (30.2)	
Grad school	1 (2.3)	
Family income		
<$10,000	14 (33.3)	
$10,000–20,000	4 (9.5)	
$21,000–30,000	6 (14.3)	
$31,000–50,000	5 (11.9)	
>$51,000	13 (31.0)	

**Table 2:** Patient clinical characteristics.

	*N* (%)	Range
Pain days in past 12 months*		0–45
0 days	5 (11.6)	
1-2 days	9 (20.9)	
3–5 days	6 (14.0)	
6–10 days	7 (16.3)	
>10 days	16 (37.2)	
Missed school days due to pain in past 12 months^+∗^		0–45
0 days	8 (19.0)	
1-2 days	6 (14.3)	
3–5 days	7 (16.7)	
6–10 days	9 (21.4)	
>10 days	12 (28.6)	
Hospitalizations in past 12 months^+∗^		0–20
0	15 (35.7)	
1-2	19 (45.2)	
3	5 (11.9)	
4	1 (2.4)	
5	1 (2.4)	
20	1 (2.4)	
ER visits in past 12 months^+∗^		0–20
0	8 (19.0)	
1-2	27 (64.3)	
3	5 (11.9)	
8	1 (2.4)	
20	1 (2.4)	

*n = 37

^+∗^ *n* = 42
